# Genome sequence and population declines in the critically endangered greater bamboo lemur (*Prolemur simus*) and implications for conservation

**DOI:** 10.1186/s12864-018-4841-4

**Published:** 2018-06-08

**Authors:** Melissa T. R. Hawkins, Ryan R. Culligan, Cynthia L. Frasier, Rebecca B. Dikow, Ryan Hagenson, Runhua Lei, Edward E. Louis

**Affiliations:** 1 Omaha’s Henry Doorly Zoo and Aquarium, Center for Conservation Research, Department of Conservation Genetics, 3701 South 10th Street, Omaha, NE 68107 USA; 2 Department of Biological Sciences, Humboldt State University, 1 Harpst Street, Arcata, CA 95521 USA; 3 Data Science Lab, Office of the Chief Information Officer, Smithsonian Institution, Washington, DC 20008 USA

**Keywords:** Climate change, Geographic information systems, Lemuridae, Single nucleotide variants, Strepsirrhine

## Abstract

**Background:**

The greater bamboo lemur (*Prolemur simus*) is a member of the Family Lemuridae that is unique in their dependency on bamboo as a primary food source. This Critically Endangered species lives in small forest patches in eastern Madagascar, occupying a fraction of its historical range. Here we sequence the genome of the greater bamboo lemur for the first time, and provide genome resources for future studies of this species that can be applied across its distribution.

**Results:**

Following whole genome sequencing of five individuals we identified over 152,000 polymorphic single nucleotide variants (SNVs), and evaluated geographic structuring across nearly 19 k SNVs. We characterized a stronger signal associated with a north-south divide than across elevations for our limited samples. We also evaluated the demographic history of this species, and infer a dramatic population crash. This species had the largest effective population size (estimated between ~ 900,000 to one million individuals) between approximately 60,000–90,000 years before present (ybp), during a time in which global climate change affected terrestrial mammals worldwide. We also note the single sample from the northern portion of the extant range had the largest effective population size around 35,000 ybp.

**Conclusions:**

From our whole genome sequencing we recovered an average genomic heterozygosity of 0.0037%, comparable to other lemurs. Our demographic history reconstructions recovered a probable climate-related decline (60–90,000 ybp), followed by a second population decrease following human colonization, which has reduced the species to a census size of approximately 1000 individuals. The historical distribution was likely a vast portion of Madagascar, minimally estimated at 44,259 km^2^, while the contemporary distribution is only ~ 1700 km^2^. The decline in effective population size of 89–99.9% corresponded to a vast range retraction. Conservation management of this species is crucial to retain genetic diversity across the remaining isolated populations.

**Electronic supplementary material:**

The online version of this article (10.1186/s12864-018-4841-4) contains supplementary material, which is available to authorized users.

## Background

**Fig. 1:**
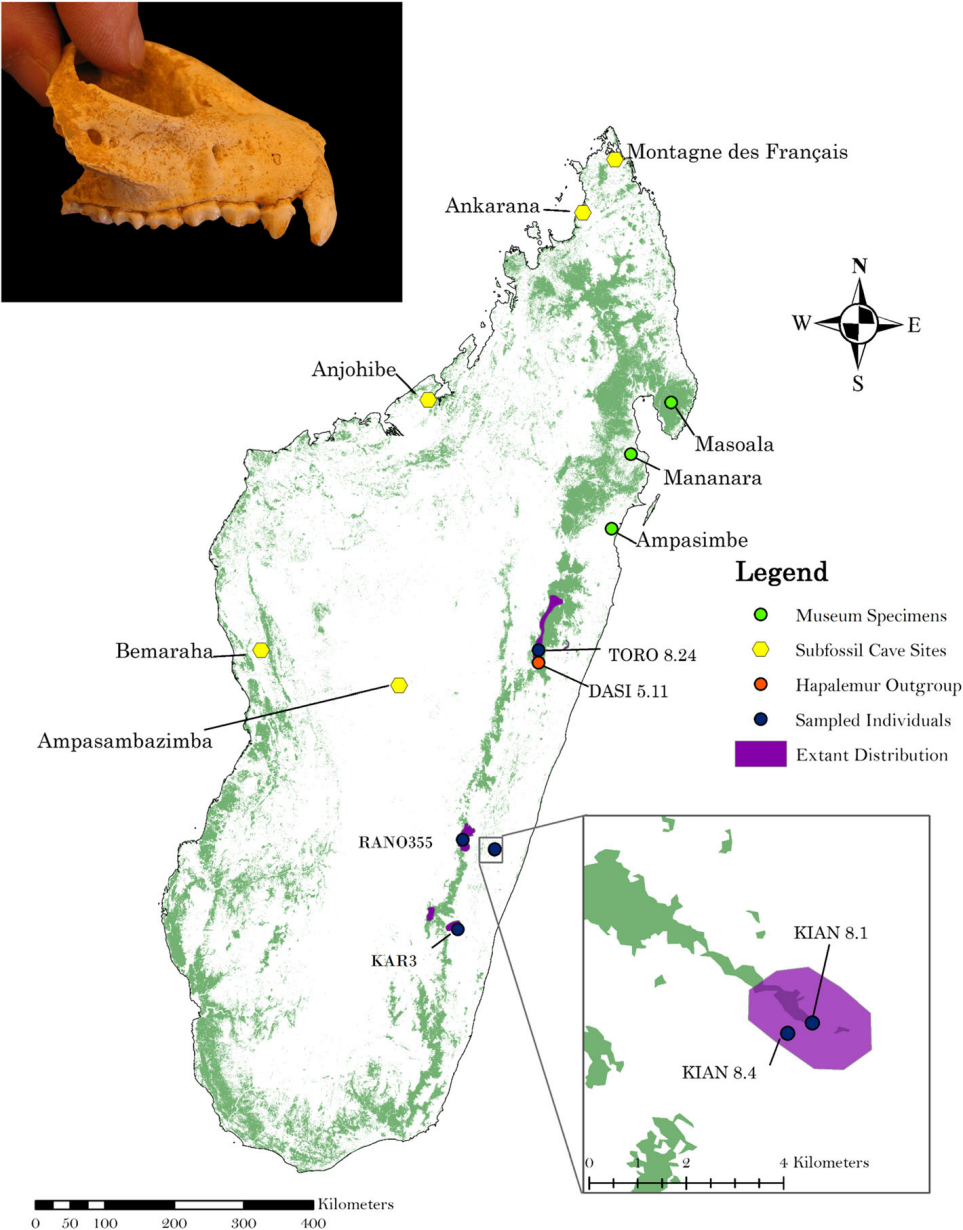
Map of Madagascar with all sequenced *Prolemur simus* plotted (KIAN8.1, KIAN8.4, KAR3, TORO8.24, RANO355) as well as the *Hapalemur* outgroup (DASI5.11). The green shading represents estimated remaining forest cover from 2005 [67], the purple is the extant distribution of *Prolemur simus* and the yellow hexagons are cave sites which have recovered subfossils of this species. *Prolemur simus* spatial data were downloaded from IUCN. 2016 *The IUCN Red List of Threatened Species. Version 2014.1*. http://www.iucnredlist.org. Downloaded on July 7, 2016. The inset photograph is a subfossil *P. simus* courtesy of Laurie Godfrey

The greater bamboo lemur (*Prolemur simus*) is an extremely rare primate, and has been listed as one of the 25 most endangered primates in the world from 2002 to 2010 [1]. *Prolemur simus* is a monotypic species of strepsirrhine (Infraorder Lemuriformes, Superfamily Lemuroida, Family Lemuridae) endemic to the island of Madagascar (Mittermeier et al. 2010). The distribution of the greater bamboo lemur historically spanned much of Madagascar, as indicated by at least five fossil sites and museum specimen localities [2–4]. Four subfossils have been radiocarbon dated to ~ 8160–2410 years before present [5, 6], but others spanning more recent time points likely exist. Currently, *Prolemur simus* is currently restricted to remaining forest patches on the eastern side of Madagascar, hundreds of kilometers south of the subfossil sites (Fig. 1; Olson et al. 2013). Some of the dated subfossils predate some estimates of colonization of Madagascar by humans, which has been estimated around 2300 years before present, ybp hereafter [7]. Additional research has recovered older evidence of human occupation in Madagascar, pushing the date of arrival to approximately 4000 ybp based on recovered stone tools and evidence of animal butchery [8–10]. The decrease in the distribution of *Prolemur simus* was likely driven by several factors potentially including climate change and aridization, human hunting and/or displacement, and more recently anthropogenic landscape modification [11].

Morphologically *Prolemur simus* is quite distinct from other species of bamboo lemurs (genus *Hapalemur*; [12, 13]). *Prolemur simus* is much larger (average weight 2.6 kg for females and 2.75 kg for males; [14]), has prominent white ear tufts, and a broad short muzzle. Like all bamboo lemurs, the greater bamboo lemur has a specialized bamboo diet, primarily consuming giant or woody bamboo (*Cathariostachys madagascariensis* or *Valiha diffusa*), which has been known to account for 72–95% of its diet [15, 16].

The greater bamboo lemur was thought to be reduced to an estimated 100–160 individuals, based on surveys spanning over two decades of research. This species has persisted in a fragmented mosaic of subpopulations isolated by deforestation, due in part, to slash-and-burn agricultural practices [11]. This lemur is found in primary forest with some degree of anthropogenic disturbance, including logged forests [14, 17]. The modification and conversion of forest to agricultural lands have far reaching effects, and are unfortunately continuing at an unabated rate in Madagascar [18–20]. Although the modern distribution of this species has expanded due to the discovery of additional populations, they remain one of the most endangered species in Madagascar [21–25]. This species is not evenly distributed in areas of occupation, rather they have been found tightly associated with *Cathariostachys madagascariensis* in Ranomafana National Park [26]. However, this bamboo species is not found at all sites where *P. simus* is distributed implying flexibility in the species of bamboo consumed (with *Cephalostachyum* cf. *perrieri* and *Valiha diffusa* as other known food sources; [16]).

At the 3rd International Technical Meeting on the conservation of the greater bamboo lemur (Ranomafana, Madagascar, August 2015), the population was estimated at over 1000 individuals, which is a milestone for the conservation of this species. Although populations are growing in some locations, the pressure on particular ecosystems remains constant, some populations have uncertain legal protection, and are threatened by mining concessions and continued forest degradation [27]. Therefore, a continued conservation effort is necessary to prevent local extinctions and allow for population recovery and stability.

Despite the dire situation of this species, no comprehensive range-wide studies have been published on the remaining genetic diversity of *Prolemur simus*, which are necessary for effective conservation programs [11]. Evaluating genetic diversity is crucial to ensure these small populations remain genetically robust, as stochastic events have been shown to cause localized population crashes [11, 28] and can inform conservation practices to assist in future translocation and reforestation efforts by including genetically diverse individuals.

Genome sequencing advancements have allowed for the sequencing of non-model organisms to occur at a rapid pace, providing unparalleled information related to conservation, evolution and immunology [29–32]. Genome studies of Malagasy lemurs have provided insight into the evolution of this unique group of primates, however as lemurs represent such a diverse adaptive radiation of primates, novel genomes are of use to a wide range of fields. The greater bamboo lemur will constitute the second species in the family Lemuridae and the first species of bamboo lemur to have its genome sequenced.

Here we sequence the genome of the greater bamboo lemur for the first time, and isolate single nucleotide variants (SNVs hereafter) from across the geographic range of this species to generate genomic resources for future conservation efforts. These genomic resources will be useful for fine scale population monitoring of the greater bamboo lemur based on non-invasively collected samples, which can be used for future population assessments. We predict to see signatures of population decline, and provide a demographic history reconstruction to date these events and determine if the major driving factors behind the population reduction in this species occurred before or after the colonization of Madagascar by humans.

## Methods

### Sample collection and genome sequencing

Lemurs were captured via a CO_2_ projection rifle with ~ 10 mg/kg of Telazol (Fort Dodge Animal Health, Fort Dodge, IA, USA) [33]. Several 2.0 mm^2^ tissue biopsies were collected from ear pinna, as well as ~ 2.0 cm^3^ of whole blood from each individual. Following sedation with Telazol, all animals allowed to fully recover prior to release at the capture site. All samples were collected with permission from Malagasy authorities (Association Nationale pour la Gestion des Aires Protégées (ANGAP), and the Ministère de l’Environnement, de l’Ecologie, de la Mer et des Forêts), exported with all national and international permits, detailed in the Supplementary Materials, and under an approved IACUC (#97–100, 12–101) through Omaha’s Henry Doorly Zoo and Aquarium. Animal handling procedures followed standards set by the American Society of Mammalogists [34].

A total of five *Prolemur simus* individuals were sequenced in this study. Individuals were selected to cover a large portion of the species’ current geographic distribution from the Torotorofotsy Ramsar site in the north to Karianga in the south. Samples also included an elevation divide, with three individuals from two low-elevation sites (Kianjavato and Karianga, 50-570 m; [11, 35] and two individuals from high elevation sites (Torotorofotsy Ramsar site [36]; and Ranomafana National Park, 900-1200 m). Samples were selected by the locations available; details are listed in Table 1.

**Table 1:** Samples included in this study, samples 1–5 are *Prolemur simus* and sample 6 is the outgroup *Hapalemur griseus*

	Sample ID	Species	Sex	Name	Location	
1	KIAN8.4	*Prolemur simus*	Male	“Mick Jagger”	Kianjavato	
2	KIAN8.1	*Prolemur simus*	Female	“Luna”	Kianjavato	
3	TORO8.24	*Prolemur simus*	Male	N/A	Torotorofotsy	
4	KAR3	*Prolemur simus*	Male	N/A	Karianga	
5	RANO355	*Prolemur simus*	Male	N/A	Ranomafana	
6	DASI5.11	*Hapalemur griseus*	Female	N/A	Andasibe	
	GPS Location		Elevation (meters asl)	Date Sampled	Genome Coverage	GenBank BioSample #
1	S21°22′33.9′′	E047°52′12.2′′	102.7	9/26/2008	152.7X	SAMN05853041
2	S21°22′40.8′′	E047°51′55.8′′	88.1	9/24/2008	8.490	SAMN05853042
3	S18°46′26.5′′	E048°25′15.9′′	981.2	2/15/2008	9.090	SAMN05853043
4	S22°25′32.5′′	E047°22′20.3′′	331.6	11/10/00	11.120	SAMN05853044
5	S21°14′30.0′′	E047°25′26.6′′	991.8	12/6/2000	8.470	SAMN05853045
6	S18°56′15.6′′	E048°25′23.9′′	973.5	5/10/2005	3	SAMN05853046

An adult male individual from Kianjavato called ‘Mick Jagger’ (sample: KIAN8.4) is monitored by our research team and was selected for deep sequencing. DNA was extracted using the phenol/chloroform/isoamyl (PCI hereafter) extraction method [37] and DNeasy Blood and Tissue Kits (Qiagen, Valencia, CA, USA). Half of a 2 mm biopsy punch of the ear pinna was used for DNA extraction as well as up to 100 μl of blood per extraction to generate 2 μg of starting DNA template. Library preparation and sequencing of this sample was done through the Hudson-Alpha Genomic Services Laboratory, (Huntsville, AL, USA) and used TruSeq style indices and tagmentation for the mate paired libraries. Several different libraries were sequenced with either 220 bp, 280 bp, or 800 bp insert lengths, as well as two mate-pair libraries (6000 and 8000 bp insert lengths) and all sequencing was done on the HiSeq 2500 platform across a total of 11 flow cells.

The genomes of four additional individuals (sequenced to a depth of ~10X genome coverage, referred to hereafter as the 10X genomes) were extracted from one eighth to one fourth of an ear tissue biopsy via PCI extraction [37]. Following DNA extraction, whole genomic DNA was quantified with a Qubit 2.0 and sonicated to an average size of 400 bp with a Covaris M220 using default settings. We then cleaned each sample with a 1X Solid Phase Reverse Immobilization (SPRI hereafter) following Rohland & Reich [38] . Following purification, samples were visualized on a Fragment Analyzer using a high sensitivity NGS kit (Advanced Analytical, Ankeny, Iowa, USA). The iTru adapters were used for library preparation following the synthesis from the UGA sequencing core [39] with Illumina library preparation kits (KAPA Biosystems) following modifications detailed in Hawkins et al. [40]. Each of the four 10X genome samples were pooled in equimolar concentrations following quantification with an ABI QuantStudio 3 and sent to HudsonAlpha for a single NextSeq 150 base pair paired end sequencing run.

A single *Hapalemur griseus* (DASI5.11) was sequenced to ~3X coverage to provide outgroup data where necessary, and to sequence the mitochondrial genome. The *Hapalemur* sample was sequenced on a partial lane of an Illumina HiSeq 2500 with paired end sequencing and an insert length of 100 bp. Library preparation was performed at the Hudson-Alpha Genomic Services Laboratory, (Huntsville, AL, USA) and also used TruSeq style indices.

### Quality filtering, genome assembly and alignment

Reads were demultiplexed by the core facility and downloaded from the BaseSpace® website (Illumina, Inc., San Diego, CA, USA). KIAN8.4 resulted in 1.36 TB of data. The 220, 280 and 800 bp insert length reads were first trimmed with Trimmomatic 0.36 (with the following options: ILLUMINACLIP:TruSeq3-PE.fa:2:30:10 LEADING:3 TRAILING:3 SLIDINGWINDOW:4:15 MINLEN:36).The two mate paired libraries were trimmed with NxTrim v0.3 using default settings [41] and subsequently trimmed with Trimmomatic under the above settings. De novo assembly was performed on the high coverage individual using SOAPdenovo v2.04 [42, 43], ABySS v.1.9.0 [44], ALLPATHS-LG v.52448 [45, 46], MaSuRCA v3.2.1 [47] and Platanus v.1.2.4 [48]. For the ALLPATHS-LG, MaSuRCA, and Platanus assemblies we thinned the raw data to reduce the overall size of the input data due to random access memory (RAM) requirements that prohibited the full dataset from being processed. The filtering was done on raw data with Trimmomatic 0.36 but the sliding window and min length requirements were modified (4, 30 and 70 respectively). The increase in quality requirements was done as we had an adequate amount of sequence data and wanted to include only the highest quality data into the genome assemblies. Assessment of genome completeness was performed with BUSCO v.2.0beta [49]. Genome assembly summary statistics were computed with QUAST v.4.3 using default settings [50]. GenomeScope [51] was used to evaluate raw read content for heterozygosity, duplication, errors and to provide an estimate of genome size. A variety of k-mer lengths were tested in Jellyfish [52] prior to input for GenomeScope.

Trimmomatic 0.36 [53] was used on the NextSeq data (consisting of the four 10X coverage genomes) and the *Hapalemur griseus* data to filter reads using the following options (ILLUMINACLIP:TruSeq3-PE.fa:2:30:10 LEADING:3 TRAILING:3 SLIDINGWINDOW:4:20 MINLEN:36). Following quality trimming sequences were visualized with FastQC 0.11.2 [54] to ensure adequate stringency in read filtration.

The 10X genomes were mapped to the KIAN8.4 genome autosome contigs using BWA v0.7.5 [55]. RepeatMasker (downloaded August 7, 2015) was used with ncbi-rmblastn-2.2.28 to blast the entire genome to prevent removal of repetitive elements, which would otherwise be removed when filtering out the sex chromosomes. The Extract Autosome pipeline, developed here (https://github.com/TheCulliganMan/extract_autosome.git), was used to remove the sex chromosomes by mapping the genomic contigs to the human X and Y chromosomes (from the HG38 assembly available at: http://hgdownload.soe.ucsc.edu/goldenPath/hg38/chromosomes/). Additionally, this pipeline removed the mitochondrial genome (GenBank Accession # NC_021959.1 was used as the reference). We annotated the reconstructed genome with MAKER v2.31.8 [56, 57], using the human genome as the reference organism for RepeatMasker (using the RepBase libraries v22.02) and Augustus v3.2.3.

### Mitochondrial genome reconstruction

To assemble the mitochondrial genome, we mapped raw reads from all samples to published mitogenomes using BWA 0.7.5. Consensus mitogenome sequences based on resulting BAM files were generated in Geneious v9.1.5. Alignments of mitogenomes were generated in MAFFT [58]. Pairwise genetic distances were calculated across the sequences generated here, along with two published mitogenomes (*Prolemur simus*: NC 021959, and *Hapalemur griseus*: NC 021950).

### Mutation rate estimation

The autosomal mutation rate was estimated for the demographic analysis. Due to the rarity of the species and few individuals in captivity, methods for calculating mutation rate following familial lines were not possible. As such, we incorporated mutation rates calculated from the grey mouse lemur (*Microcebus murinus*) as to our knowledge they represent the closest relative with a published autosomal mutation rate [32].

### Generation time estimation

The estimated age for *Prolemur simus* to reach sexual maturity is 3.5 years [14]. The latest age of reproduction is unknown in this species, but animals from a long-term monitored site were still breeding until 12 years old, implying 12 years may not be the oldest age at which this species reproduces (unpublished data). The closely related ring-tailed lemur (*Lemur catta*) has been known to live to 18–20 years in the wild and reproducing beyond the age of 13 years, although precise metrics are not available [59]. Based on the data from both the greater bamboo lemur and the ring-tailed lemur we estimated the median generation time as 10 years, based on field observations of females which are minimally 10 years old and continue to successfully raise offspring.

Summary statistics for the autosomal variants were produced from each vcf file with the bcftools ‘stats’ command [60]. Also, we limited the input data for KIAN8.4 to make the results comparable to the 10X genomes. We used the largest number of sequences from the 10X individuals (in this case KAR3) to cap input sequences for KIAN8.4. Additional details regarding the command line usage and calculation of mutation rate can be found in the Supplementary Materials.

### Demographic history

In order to understand the demographic shifts in this species through time we used the program PSMC v0.6.5 [55]. This program has been widely used in non-model organisms to understand shifts in effective population size through time by incorporating mutation rates, generation times and genomic sequence data. We used PSMC with the following parameters: psmc -N25 -t15 -r5 -p “64*1”. We also tested the high coverage genome for false negative rates where we mapped twice the amount of data to determine the number of variants lost due to coverage biases as suggested in the PSMC documentation [61]. We acknowledge the depth of coverage for estimating demographic history via a PSMC analysis is less than ideal following the recommendations of Nadachowska-Brzyska et al. [62], but compensate by incorporating false negative rate. In addition, we generated a demographic history reconstruction using the full dataset for the high coverage genome.

In addition to the PSMC plots of *P. simus*, we generated plots from the published genome of *Propithecus coquereli* and *Microcebus murinus* to compare demographic histories across three lemurid families. A single HiSeq lane of data was downloaded from NCBI’s Short Read Archive for each individual (*P. coquereli* Accession: SRX763489, and *M. murinus*: SRX767314). The published genome scaffolds for each individual (*P. coquereli* and *M. murinus*) were used to map the raw reads, to determine the number of variants as detailed above. The divergence dates for the two were estimated at 2.27 MYA (split between *P. coquereli* and *P. tattersalli*) and 5.89 MYA (split between *M. murinus* and *M. griseorufus*), following the dated tree in [63]. The generation time was estimated at 10 years for the *Propithecus* and three and four and a half years were both tested for *Microcebus*. It is important to note that the *M. murinus* sample was from a captive bred individual with unknown genomic effects on the sample. The demographic reconstruction of this sample in particular should be interpreted with caution. Additional details regarding the PSMC reconstruction on the *Propithecus* and *Microcebus* can be found in the Supplementary Materials.

### SNV discovery

We identified SNVs for subsequent F_ST_ and PCA analyses of population structure as well as for use in future genotyping efforts. SNVs were identified across each recovered genome using samtools (mpileup option; [55]) and bcftools (call option; [60, 64]). For the high coverage individual (KIAN8.4) we used the same number of reads mapped for the demographic history reconstruction to prevent an inherent bias in the number of identified SNVs. We calculated nucleotide diversity across windows of 100 kb using samtools (window-pi option). These values were plotted across scaffolds as chromosomes have not been characterized in this species. To isolate genome wide polymorphisms, we used the pipeline from Genome Analysis Toolkit v3.6 [65] to identify shared SNVs following the commands listed on: https://github.com/TheCulliganMan/snp_pipeline/blob/master/snpPipeline.py. Once high quality SNVs were identified, variants were spaced (minimally 50,000 bp) to reduce the possibility of linkage between sites, and linkage disequilibrium was calculated using SNPRelate [66]. Sites were also scanned for variation, and sites with at least three genotypes (for example; AA, AC, CC) were included in a principal component analysis in R with the package ggfortify v0.2.0 (autoplot and prcomp commands). We used this subset of SNVs to calculate F_ST_ values across elevations and geographic distances with VCFtools [60]. A variety of comparisons were performed between geographic locations to identify the strongest signatures of differentiation. In addition to the SNV discovery, we compare genome wide heterozygosities (GWH hereafter) of the greater bamboo lemur to several published genomes. This was calculated by dividing the estimated genome length by the number of identified variants. A limited number of genomes were compared where data were available.

### Historical range estimation

The modern distribution of the greater bamboo lemur is a fraction of its former extent based on fossil sites and records of museum specimens (Fig. 1). IUCN spatial data were downloaded for the extant distribution of *Prolemur simus*. We also added cave sites and locations from museum records. All of these points were merged in ArcMap v10.4.1, and we estimated a minimum convex polygon. We then clipped the range based on various maps of forest cover [1950’s and 2005, from [67]. Contingent on the rates of deforestation we buffered the edges of the forest fragments with a variety of distances to emulate forest cover pre-human colonization using the 1950’s forest cover map (500 m, one and five kilometers). Data for these estimates were obtained from [67] . This study showed a 40% decrease in forest cover from the 1950’s-2005. In the 1950’s, only 27% of Madagascar was covered by forest, of which a half to two thirds may have already been destroyed by humans. We calculated the potential historical occupation for the greater bamboo lemur based on the estimates from the 2005, 1950’s raw and 1950’s buffered areas.

## Results

### Genome coverage

The approximately 2.39 gigabase genome was sequenced to an estimated depth of 152.7X with a scaffold N50 of 2.7 megabases from what we deemed our best assembly recovered from MaSuRCA [47]. We evaluated each assembly by comparing the number of contigs, length of contigs, and length and number of scaffolds recovered and compared that to the estimated genome length and how many complete genes were recovered from BUSCO. Approximately 68.85% of reads were retained after quality filtering (Additional file 1) The stringency of the quality filtering was increased substantially due to the amount of sequence available. Details of the performance of the other assemblers can be found in Additional files 2 and 3. We assessed genome completeness with BUSCO v.2.0beta, and recovered between 81 and 97% of mammal genes (details in Additional file 4). GenomeScope [51] was run on quality trimmed reads to provide an estimate of genome wide heterozygosity and repeat content, which resulted in 0.622 and 3.4% respectively. A length estimate of 1.8 gigabases and error rate of 0.08% were also recovered. Plots of these results are in Additional file 5.

To map the 10X genomes to the KIAN8.4 genome we removed contigs containing the X and Y chromosomes using Extract Autosome as detailed above in the Methods section. A total of 211,576 contigs were generated in the MaSuRCA assembly. After repeat masking and extracting the X and Y chromosomes plus the mitochondrial genome 9470 contigs were removed, or approximately 1.2% of the contigs, leaving 202,106 contigs representing the autosome. Based on base pair counts, approximately 10.54% of the contigs were removed. Specific details of the autosome extraction are found in Additional file 6. Details of the MAKER annotation can be found in Additional file 2. We did not analyze the sex chromosomes for the greater bamboo lemur since the closest relative with a characterized X chromosome is the mouse lemur (*Microcebus murinus*), which diverged from the greater bamboo lemur over 30 million years ago. To characterize the bamboo lemur sex chromosomes long read sequencing would be beneficial.

The four 10X genomes recovered 389 gigabases of data from a single NextSeq run. Each sample recovered an average of 231 million reads (ranging from 211,625,646 to 285,331,224 reads, detailed in Additional file 7). Approximately 5% of reads were discarded during quality filtering. The coverage of the four 10X genomes recovered an average depth of 9.3X ranging from 8.48 to 11.12 (Table 2).

**Table 2:** Mapping results from the four low coverage genomes. Mapping was performed with BWA, and identification of variants was done using samtools

	Autosome			
	average depth	Standard deviation	number of reads mapped	number of variants identified
KIAN8.4	10.910	18.78	100,810,101	4,841,672
KIAN8.1	8.490	14.56	74,521,374	6,988,664
TORO8.24	9.090	16.81	77,961,703	7,449,843
KAR3	11.120	18.84	97,754,623	7,427,674
RANO355	8.470	15.14	73,860,842	6,813,236

### Autosome extraction

We believe removing 10.54% of contigs is an acceptable amount of sequence to remove as the X chromosome is estimated to contribute to 5% of the human genome, and the Y ~ 1% in humans, totaling ~ 6%. While we removed an additional ~ 4% we cannot rule out that the greater bamboo lemur has sex chromosomes of different lengths than humans. We would rather err on the side of removing extra to prevent X and Y contigs within the autosomes. We also removed 15 contigs when we extracted the mitochondrial genome.

### Mitochondrial genomes

The published *Prolemur simus* (GenBank Accession: NC_021959) mitogenome was used to map filtered reads. Reads were mapped with BWA v.0.7.5 [55], and BAM files were loaded into Geneious v.9.1.5 to generate consensus sequences and summary statistics (Additional file 8). An average of 233,763 reads were mapped, resulting in an average coverage of 1589.2X. We also included two outgroup mitogenomes, a published *H. griseus* mitogenome (GenBank Acession: NC 021950) and another *H. griseus* from a low coverage genome sequenced in our laboratory (Table 1). Genetic distances between individuals were relatively low, with TORO8.24, from the northern part of the species’ current range, approximately 1% more distant than those in the south. Interestingly, the samples two southern sites, Karianga and Ranomafana, had nearly identical mitochondria with only two substitutions across the entire mitogenome. The mitochondrial similarity may indicate a population bottleneck and/or founder effect between the Karianga and Ranomafana populations.

### Demographic history reconstructions

We mapped reads from a subset of a HiSeq lane (800 bp insert library: 102 million (102,026,022) trimmed reads to match the highest number of reads from the low coverage data, KAR3) to the consensus contigs from the KIAN8.4 genome without the X and Y chromosomes and mitogenomes to detect heterozygous sites within KIAN8.4. This resulted in 100 million (100,480,379) sequences mapping, or ~ 98% of reads, to an average depth of 11.08X. From here, we counted the total number of variants across the entire genome with samtools ‘mpileup’ command, followed by bcftools ‘call’ (−m was used to capture rate variants). This resulted in 5,042,169 autosomal variants (including indels). We implemented mutation rates calculated from captive grey mouse lemurs (*Microcebus murinus*) as no other phylogenetically proximate species have robust genomic mutation rate calculated [40].

We also mapped reads from single sequencing lanes for the two published lemur reference genomes to provide a broader demographic reconstruction for lemurs. From these files, we counted variants which summed 6,614,286 and 6,808,109 for *Propithecus coquereli* and *Microcebus murinus,* respectively. The same autosomal mutation rate was used for these species and generation time estimates are detailed in the Supplemental Materials. Read mapping resulted in average coverage of 8.83X and 12.44X for *P. coquereli* and *M. murinus* respectively; additional details are presented in Additional file 9: Table S6 and plots are shown in Additional file 10.

**Fig. 2:**
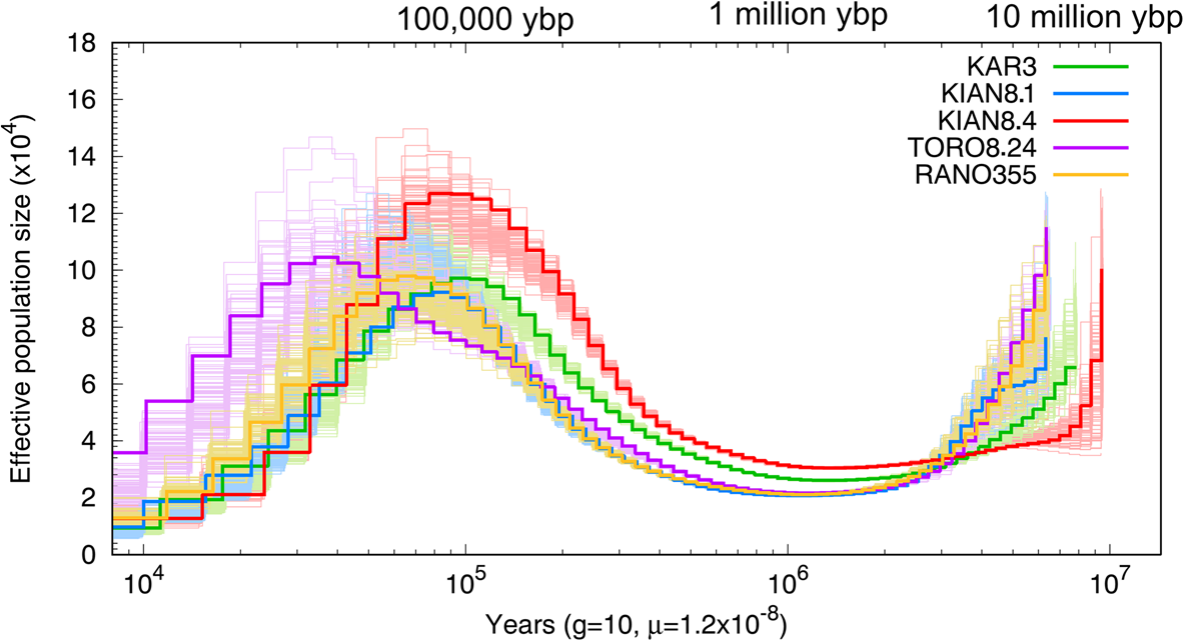
Demographic history of all *Prolemur simus* individuals. Dark lines of each color represent the whole genome reconstruction of the demographic history, and each of the lighter colors represents 100 subsampled bootstrap replicates for each individual. The legend on the top right identifies the colors of each individual and a non-log timescale is presented on x-axis. This plot was corrected for the 12% false negative rate (FNR) estimated from the high coverage genome

Following the suggestions by Li, we tested for the rate of false negatives in low coverage genomes by mapping twice the number of reads and identified variants from KIAN8.4 [61]. We recovered 5,733,824 SNVs, or 691,655 more than the low coverage equivalent iteration of mapping. We used this to calculate a false negative rate (FNR hereafter) of 12%, which was applied to all samples for correction in PSMC. The resulting plot (after correcting for FNR) can be found in Fig. 2. As precise details of the life history of *Prolemur simus* are not available, we used a generation time of 10 years for this species as detailed in the Methods section.

We recovered a significant and severe decline in range-wide populations of *Prolemur simus* (Fig. 2). Our demographic history reconstructions reveal an increase in effective population size from around 10 million years ago until approximately 100,000 ybp. The greatest population size estimated from each individual differed between the southern individuals (referred to as such hereafter, and includes KAR3, KIAN8.1, KIAN8.4 and RANO355) versus the single northern sample (TORO8.24). In the southern samples, we recovered the highest population estimates of approximately 1000,000 individuals around 60–90,000 ybp. In the northern sample, we estimated a peak of right around 1000,000 individuals around 35,000 ybp. The southern samples, underwent a population crash starting 80–50,000 ybp. The northern sample had a gentle increase from about 200,000 ybp until approximately 35,000 ybp, after which the population decreased significantly. After these respective crashes between the northern and southern samples, the Kianjavato samples averaged an effective population size of approximately 110,000 individuals (from over 1000,000). Karianga resulted in a population of approximately 92,000, while Ranomafana and Torotorofotsy were reduced to ~ 62,000 and ~ 170,000 respectively. These population estimates are based on the PSMC plot generated after accounting for FNR (Additional file 11), but are based on 10X genome coverage and as such should be interpreted with caution. Deeper sequencing could reveal additional polymorphisms not detected here. We do not recommend using the above effective population size estimates for detailed analysis, as the differences may not reflect accurate biological changes through time and estimates may be altered with deeper genome sequencing. In order to evaluate how much the coverage affected the demographic reconstructions reported here, we generated a PSMC plot for the KIAN8.4 sample using all the data for the approximately 150X genome. When comparing the results with between the KIAN8.4 full coverage and KIAN8.4 ~10X genome we recover approximately a 2–7% differences in the estimated effective population sizes. The PSMC plot for the 150X coverage genome can be found in Additional file 12.

We refrain from speculation about the demographic history of the greater bamboo lemur from present time to ~ 20,000 ybp as PSMC is not accurate in this time period. Additionally, the PSMC results recovered here represent a general population trend through time, and across the range of this species, for finer scale reconstructions, and to determine the extent of isolation between the northern and southern samples, deeper sequencing efforts should be performed. These dramatic population crashes represent an average decline of 89% between the maximum effective population size estimates (60–90,000 ybp) to ~ 20,000 ybp.

### SNV identification

**Fig. 3:**
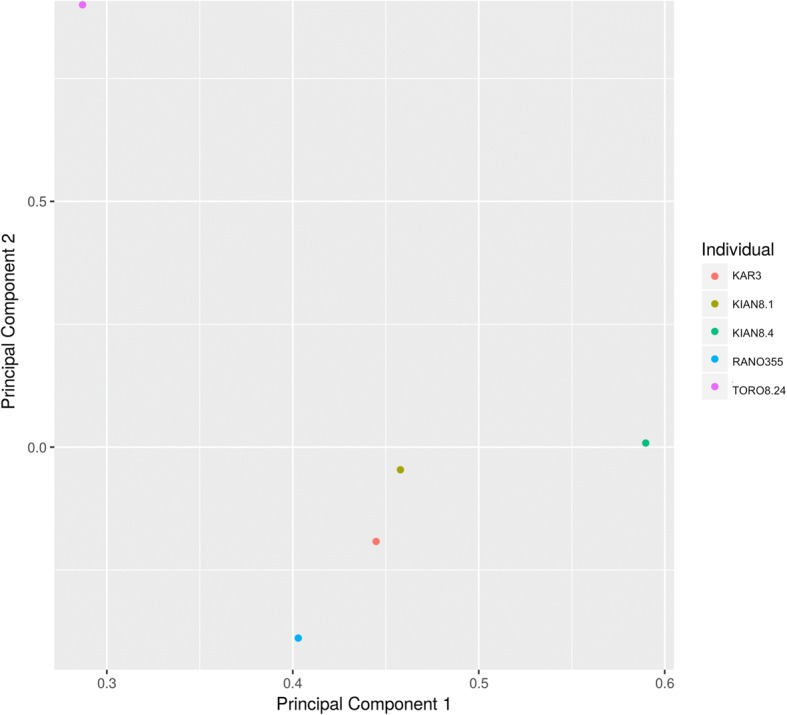
PCA of a set of ~ 19,000 SNVs. Sample names are noted in the legend, and weight of the different axes are shown. Principal component 1 and 2 are shown which accounted for 47 and 18% of variation, respectively

Across all genomes, we identified 33 million (33,331,199) SNVs for *Prolemur simus*. We identified 7,585,751 variants across all five samples. Details of the VCF filtration (done via VCFtools) can be found in Additional file 2. When we applied spacing parameters to the SNVs (50,000 bp minimally between included polymorphisms) we reduced the dataset to 152,361 SNVs. We evaluated this set of SNVs for linkage in SNPRelate and recovered 118,609 which had less than 0.2 r^2^ values. To further reduce this dataset for practical reasons to apply to subsequent population genomics studies, and to include highly variable markers, we excluded SNVs which did not have at least one individual homozygous for both variant alleles (e.g., a SNV would be included if there were CC, CG and GG as alternates, but if only CC and CG were found it would be excluded). A set of nearly 19,000 SNVs were analyzed via PCA (Fig. 3). The subset of 18,798 SNVs were tested for signals of linkage using VCFtools (−-geno-r2 flag). We recovered 2022 SNVs with an r^2^ of 1 (indicating those sites were perfectly linked), 1137 which recovered an r^2^ ranging 1- > 0.75, 995, 0.75- > 0.5, 4583, 0.5- > 0.25, 4277, 0.25- > 0.1, 5784, between > 0.1–0, and of those 715 had an r^2^ of 0 (indicating these SNVs were in equilibrium). We want to caution over speculation related to the extent of linkage as this was across only five individuals and two of which were from the same population. We also calculated F_ST_ values when separating the samples in two criteria: by elevation (TORO8.24 and RANO355 together versus KAR3, KIAN8.4 and KIAN8.1) and along a north-south gradient (TORO8.24 separated from all individuals) which resulted in a weighted F_ST_ of 0.015 and 0.144, respectively. We also recovered pairwise F_ST_ values across the southern population, which recovered the following results; Kianjavato-Ranomafana (KIAN8.4 and KIAN8.1 were both included for all Kianjavato comparisons) 0.05, Kianjavato-Karianga 0.038.

After searching the literature GWH were compared between the following lemurs; greater bamboo lemur, coquerel’s sifaka and the grey mouse lemur, all of which the variant counts were generated here (as well as the estimated autosome length for the greater bamboo lemur). Additionally, we compared GWH to the African wild dog genome [68], and cheetah genome [69], as they represent African carnivores with varying degrees of genetic diversity. The average GWE for the greater bamboo lemur was 0.0037, 0.0033% in the coquerel’s sifaka, 0.0039% in the grey mouse lemur, 0.0017% in the cheetah and 0.0074% in the African wild dog.

### Historical range estimation

**Fig. 4:**
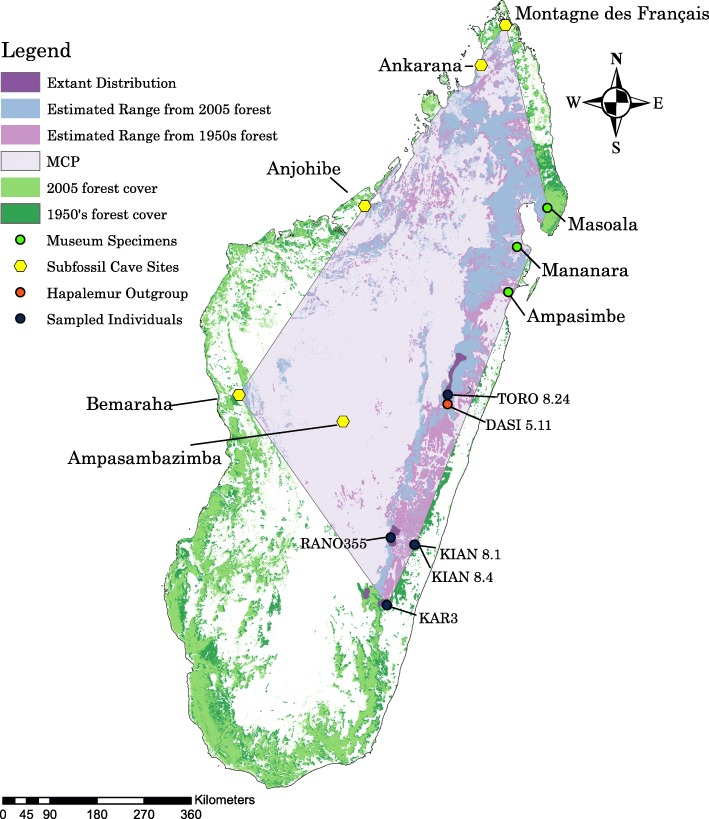
A rough estimate of the historic range of *Prolemur simus* incorporating cave sites which contained subfossils, modern distribution data and museum specimens. The estimated home range shown here was generated from the 2005 forest cover of Madagascar. A minimum convex polygon (MCP) was drawn between the locations of subfossil, museum, and contemporary *Prolemur simus* occupation, and the calculated area was restricted to forested regions within the MCP. The outgroup was a *Hapalemur griseus*

Based on the distribution data obtained from IUCN, we calculated the extant distribution of *Prolemur simus* across a handful of forest patches totaling 1711.74 km^2^. If we estimate the range of *Prolemur simus* between the subfossil and museum specimen sites as well as modern records using a minimum convex polygon we recover an area of 44,259.65 km^2^ at the current forest extent (based on the 2005 forest cover map, see Fig. 4). If we use the forest cover from the 1950s the potential distribution was 80,375.37 km^2^. Since we know humans degraded the forest prior to 1950, we extended the forest by buffering the edges and recovered estimated ranges of 96,144.1 km^2^, 108,003.45 km^2^, and 163,223.84 km^2^ respectively for the 500 m, one and 5 km buffers, which would depend on the extent of anthropogenic deforestation. The estimated range based on the 1950s forest cover can be found in Fig. 4. Maps containing the buffered regions are presented in Additional file 13.

## Discussion

### Genome resource

To our knowledge, we generated the most complete genome from the strepsirrhine family Lemuridae to date, comparable in quality to one of the two available lemur reference genomes (*P. coquereli* v1.0). We tested five de novo assembly programs and recovered a robust (~ 150X) genome. In the genome, we identified over 5 million SNVs, and over 31 million additional SNVs across the four 10X genomes. A single low coverage *Hapalemur griseus* was sequenced to approximately 3X, and when this species was mapped to *Prolemur simus* we identified over 16 million SNVs between the two genera. From the shared SNVs within *P. simus,* we filtered down to ~ 19,000 variable SNVs for population genomic studies. We show preliminary evidence for geographic isolation between northern and southern samples. These high quality SNVs will benefit future research efforts into population subdivision in *P. simus*.

From these sequencing efforts, we estimate the genome of *Prolemur simus* to be approximately 2.39 gigabases in length, recovered from the MaSuRCA assembly. The GenomeScope results yielded a shorter length of ~ 1.85 gigabases, which is short for a mammalian genome. This may be due to the relatively high amount of duplicates identified in GenomeScope. This genome adds to the two high-quality lemur reference genomes available and represents a third lemur family with a high-quality genome.

The MaSuRCA assembly recovered over 97% of single copy orthologs included in the mammal gene set via BUSCO v2.0beta. Additionally, the estimated genome size was closest to the expected length (2.39 gigabase) than from the other tested assemblers. For reference, the *Microcebus* and *Propithecus* genomes have been estimated at 2.44 and 2.80 gigabase, respectively. GenomeScope provided descriptive statistics on the overall genome composition (with a k-mer of 40), which recovered 0.622% heterozygous sites, at an error rate of 0.0803%, with 2.51 duplicate molecules (see Additional file 5).

F_ST_ values were calculated to demonstrate the similarity between genomes from the different sampled populations. The strongest signal of differentiation occurred along a north to south gradient, separating Torotorofotsy from all other populations. This may reflect an artifact of drift since the populations are also the most geographically separated. The low elevation populations Kianjavato and Karianga were most similar, despite the closer proximity of Ranomafana to Kianjavato.

In addition to the above metrics, we compared GWH between the greater bamboo lemur and a handful of other published genomes. The average GWH in *P. simus* was 0.00365%, compared to 0.00329% in the published sifaka genome. The *M. murinus* published genome recovered 0.00338% GWH. When we compare outside of lemurs the cheetah genome, a species known to have undergone severe population bottlenecks recovered a 0.00168% GWH, and 0.00738% in the African wild dog genome [68, 69]. All of the lemur genomes we calculated GWH had similar levels of H_e_. The cheetah genome had about half the amount of GWHs compared to lemurs, and African wild dogs had about twice the level of GWHs compared to all lemurs.

### Demographic history

The dramatic decline in effective population size is an important finding for this species as this occurred during the Pleistocene, a time of global climate change. Our data provides genomic signatures where there is currently a lack of paleontological information. Climate inferences from more recent time points have been mapped, as detailed in [7] . We also generated PSMC plots for both published reference lemur species, and recovered a remarkably similar crash in the coquerel’s sifaka during the Pleistocene (Additional file 10). The grey mouse lemur did not show a crash, rather a slight expansion through time. The black and blue-eyed black lemurs, Genus *Eulemur*, [70] also showed a crash in the population ~ 700,000–1 million years ago, which the authors inferred as an aridity shift [71]. The crash in the black and blue-eyed black lemurs corresponded to an approximate 50% decrease in effective population size. In our brief evaluation of the *Microcebus* genome, we recovered an expansion in the population of the mouse lemur and a population decline of 53% in the coquerel’s sifaka. Additional details related to these calculations can be found in Additional file 2.

PSMC estimates of effective population size circa 20,000 ybp (when estimates are still accurate in the program) were ~ 109,000 individuals (Additional file 11), compared to the modern census population estimates of around 1000 individuals (IUCN Red List, http://www.iucnredlist.org/details/9674/0 Accessed April 18, 2017). This secondary decline represents a 99% decline from the historic population estimates to the modern census (which is obviously an overestimate of the extant effective population size). If we estimate the population crash from the highest estimates in PSMC around 60–90,000 ybp the reduction is 99.9% (reduced from approximately one million individuals to 1000).

We postulate that the two arid adapted species (*Eulemur* and *Propithecus)* underwent a less dramatic decline, possibly because they were previously adapted to a drier climate. *Microcebus murinus* is also distributed in the west and south, but due to the large range and unknown origin of the sample (as well as possibly genomic consequences of captive breeding) we refrained from further speculation for this species. Fine scale estimates of demographic history for these species will be useful to better characterize changes in lemur effective population sizes through the Pleistocene Epoch. Due to the limitations of our sampling scheme, as well as the available programs, more recent demographic reconstructions are not well resolved in this species, and represents an avenue of future research.

### Historical range contraction

Estimates put the rate of deforestation in Madagascar around 40% since the 1950’s [67], which does not account for the half to two thirds reduction in forest cover pre-human colonization. The greater bamboo lemur is assumed to have decreased in population size (as documented by subfossils around Madagascar), but for the first time we quantified the timing and scale of this decline.

The range of this species has retracted from its original distribution from the northernmost tip of Madagascar (Ankarana and Montagne des Français) down the southeast (extant distribution and museum locations), across the central highlands (Ampasambazimba), and to caves along central western Madagascar (Bemaraha and Anjohibe). The current distribution spans 1711.74 km^2^. A very rough estimate of the potential historical distribution was calculated to equal 80–100,000 km^2^. The extant distribution covers only ~ 1.5–2% of this potential historical distribution. Our estimates of the historical distribution match with previous estimates of range collapse in this species [11].

It is likely that the change in climate affected the distribution of the bamboo species consumed by the greater bamboo lemur and thus led to the extirpation from vast portions of the island. Bamboo surveys found the genus *Valiha* at one subfossil site suggesting it may have been the food source at these locations [16]. *Valiha diffusa* is the dominate food source for the *P. simus* in Kianjavato [14], however at higher elevations *Cathariostachys madagascariensis* is recorded as the most common food item [15], possibly because *Valiha* is not distributed at higher elevations.

While PSMC is unable to reconstruct the recent demographic history, we have evidence from subfossils and museum records that *P. simus* persisted in locations far beyond their current distribution until the nineteenth century. Museum records indicate three specimens from far northeast Madagascar, near the Bay of Antongil (estimated around Masoala on Fig. 1), Mananara and Ampasimbe. These specimens were collected from northeast Madagascar between 1870 and 1913 [24]. Modern records of *Prolemur simus* range from: Zahamena in the north (inferred from feeding traces; [23] to Andringitra in the south [72]. Our estimate of the historical distribution of the greater bamboo lemur resulted in only 3.87% occupancy of their potential habitat based on the most recent forest cover maps (2005). If we use the 1950’s the estimated range, the estimated area of occupancy is ~ 2.1%. If we add a forest buffer around the 1950’s map, the area occupied is reduced to a mere 1.05–1.78% (see Additional files 13 and 14). Ecological niche modeling would likely improve upon the crude estimates provided here by incorporating spatial use of forests by this species, and locations of bamboo thickets within forest patches.

Based on the historical and modern range information, plus the demographic reconstructions we can deduce that humans likely caused a secondary decline in this species. Additionally, this species was distributed far north of the current limit prior to the past 150 years as documented by museum specimens [2].

### Extinctions and climate change in the Pleistocene

A variety of hypotheses have been proposed to explain the massive extinctions during the Holocene. These range from human mitigated fire [73], climate change and drought [74], blitzkrieg hunting [75, 76], disease [77], and synergy (which connects climate change to human impacts; [78] . A more complete account of these factors are detailed in [6]. In Africa, climate change through the Pleistocene and Holocene led to aridization, and expansion of the Sahara desert [79]. However, island systems likely function differently, and Madagascar was not included in the previously mentioned analyses. Despite the lack of modeling resolution, it is probable that humans increased the magnitude of extinctions in Madagascar over the past 2300 years of occupation [7]. The population crash in the greater bamboo lemur long predates the arrival of humans, although they underwent an additional decline following human colonization.

The population crash identified in the demographic reconstruction is not unique to the greater bamboo lemur as additional lemurs (*Eulemur* and *Propithecus*) recovered declines during the same time period. The aridification of Madagascar has been suggested as the driver in the decline of *Eulemur* [70], which occurred around one million years ago and appears to have affected both diurnal and nocturnal species distributed across three extant families. The decline in effective population sizes across four genera, which include three lemurid families, may demonstrate an island-wide decrease in lemur densities. Modeling has also been done for the Tattersall’s sifaka (*Proithecus tattersalli*), and results indicate this species has undergone a population decline predating human colonization [80].

### Conservation implications

Combining all lines of evidence, we recover a dramatic and prolonged decline in the greater bamboo lemur. This species is the sole member of the genus *Prolemur*, and faces a dire conservation situation. Historically this species was likely widely distributed and common around Madagascar. In the Ankarana cave systems, *P. simus* was so abundant that researchers had to take care when walking to avoid stepping on the hundreds of greater bamboo lemur bones [6]. The absence of this species near Ankarana and the Masoala peninsula where there are still large tracts of forest may imply human-related extirpation events, anecdotally in certain areas this species is hunted for food.

The greater bamboo lemur is a Critically Endangered species, which has undergone minimally two major population declines; one natural in origin (likely related to climate shifts), and the second anthropogenic. Continued research efforts are necessary to better characterize and understand population densities, and seek protected status for forests where this species is found. In addition to the range-wide declines, we recovered genetic signatures of differentiation between the northern and the southern samples. This may imply a long period of isolation prior to range contraction, and possibly due to a river barrier (Mangoro River). Additional studies of populations surrounding this river may provide greater insight to the degree of latitudinal differentiation in this species. Long-term conservation plans should be implemented to ensure the survival of this unique species with the goal of retaining the remaining genetic diversity. The genomic resources generated here will be useful for characterizing population level genetic diversity, and allow for the implementation of conservation management plans.

## Conclusions

We provide one high coverage genome for the greater bamboo lemur (*Prolemur simus*) as well as five lower coverage genomes from across the extant range of this species. We identified ~ 152,000 SNVs between the five individuals, and used ~ 19,000 SNVs for a principal component analysis which recovered a stronger signal separating individuals across a north to south gradient, possibly isolated across the Mangoro River. A demographic reconstruction using the program PSMC identified a significant decrease in the effective population size beginning 60–90,000 ybp. Following this decrease (and beyond inferences from PSMC) a second likely-anthropogenic decrease has reduced the population to a census of approximately 1000 individuals. This represents an 89% decrease from the estimates approximately 20–40,000 ybp (where PSMC estimates are still considered accurate) and a 99.9% decrease from the peak estimate of effective population size. This species had similar levels of GWH as other lemurs where comparisons were possible. Additionally, this species is listed as Critically Endangered and requires immediate and continual conservation attention to preserve genetic diversity in the highly fragmented forests this species resides.

## Additional files

Additional file 1: Quality filtering for KIAN8.4 ‘Mick’ Genome. Section A details the results from Trimommatic for the short insert length libraries. Section B details the results of the NxTrim combined with Trimmomatic for the mate-pair libraries. (DOCX 91 kb)

Additional file 2: Supplementary Materials detailing permits, genome assembly, MAKER annotation, Demographic history reconstruction- *Propithecus* and *Microcebus*, VCF Filtration. (DOCX 31 kb)

Additional file 3: Comparison of scaffolds and contigs across different assembly programs. The top row shows the largest 1000 and 10,000 scaffolds across each program (from left to right) and the bottom row displays the longest 5000 and 50,000 contigs. ALLP in the figure caption represents the ALLPATHS assembly. (JPG 3784 kb)

Additional file 4: BUSCO v2.0beta assessments of genome completeness for the five different genome assemblies, using the ‘mammal’ set of 4106 genes. (DOCX 46 kb)

Additional file 5: GenomeScope Profile of the KIAN8.4 high coverage genome. (TIFF 2484 kb)

Additional file 6: Autosome Extraction Results from the KIAN8.4 MaSuRCA contigs. (DOCX 51 kb)

Additional file 7: Quality Filtering Stats from 10X genomes using Trimmomatic v0.36. (DOCX 52 kb)

Additional file 8: Statistics from mitochondrial genomes from the five *Prolemur simus* and one *Hapalemur griseus*. Coverage statistics as well as the GenBank Accession numbers are listed. (DOCX 61 kb)

Additional file 9: Results of read mapping of published lemur reference genomes. Accession numbers, coverage, standard deviation, variants and origin of tissue are listed below. (DOCX 46 kb)

Additional file 10: PSMC plots of the two lemur reference genomes. The top two images show the *Microcebus murinus* genome reconstructions with 100 bootstrap replicates. Image ‘a’ applied a three-year generation time, ‘b’ incorporates a 4.5 year generation time as detailed in (Yoder et al., 2016). The third image, ‘c’, is the demographic history reconstruction of *Propithecus coquereli*. The population crash shown in ‘b’ occurred during the time period where PSMC reconstructions are inaccurate and as such we refrain from making inferences from that graph. (TIFF 1614 kb)

Additional file 11: PSMC estimates of effective population size following Pleistocene bottleneck. (DOCX 35 kb)

Additional file 12: PSMC plot from the 150X genome reconstruction of KIAN8.4. Note the scale of the y-axis in this graph is not the same as the PSMC plot from the main text. (PDF 174 kb)

Additional file 13 A comparison of the effect of buffered edges around the 1950’s forest cover. The minimum convex polygon is shown in pale yellow, and the three different buffered distances are shown in light blue, peach and olive respectively. The degree of deforestation would affect which buffered distance is most likely for this species to have historically occupied. (PDF 5673 kb)

Additional file 14: Historical range calculations based on varying levels of forest buffer. Forest area is given in square kilometers. (DOCX 39 kb)

## Abbreviations

Bp: Base pair
GWH: Genome wide heterozygosity
H_e_: Heterozygosity
Kg: Kilogram
MYA: Million years ago
SNV: Single nucleotide variants
Ybp: Years before present
