# Supplementary material for: Genome sequence and population declines in the critically endangered greater bamboo lemur (Prolemur simus) and implications for conservation
Source: BMC Genomics. 2018 Jun 8;19:445. doi: 10.1186/s12864-018-4841-4 (PMC5994045; doi:10.1186/s12864-018-4841-4)
Supplement: Supplementary file 1 — Quality filtering for KIAN8.4 ‘Mick’ Genome. Section A details the results from Trimommatic for the short insert length libraries. Section B details the results of the NxTrim combined with Trimmomatic for the mate-pair libraries. (DOCX 91 kb) [file 12864_2018_4841_MOESM1_ESM.docx]

Table S1. Quality filtering for KIAN8.4 'Mick' Genome. Section A details the results from Trimommatic for the short insert length libraries. Section B details the results of the NxTrim combined with Trimmomatic for the mate-pair libraries.

| A. | Insert Length (bp) | Number of HiSeq Lanes | # raw read pairs | Both surviving | Forward only surviving | Reverse only surviving | Dropped | Remaining |
| --- | --- | --- | --- | --- | --- | --- | --- | --- |
| **Trimmomatic** | 220 | 4 | 1,278,960,204 | 612312354 (47.88%) | 182714331 (14.29%) | 103785519 (8.11%) | 380148000 (29.72%) | 70.28% |
|  | 280 | 3 | 397,393,878 | 242204071 (60.95%) | 54246463 (13.65%) | 35506002 (8.93%) | 65437342 (16.47%) | 83.53% |
|  | 800 | 2 | 322,777,831 | 107757443 (33.38%) | 39428337 (12.22%) | 23088389 (7.15%) | 152503662 (47.25%) | 52.75% |
|  |  |  |  |  |  | Total reads after quality trimming: | | 1,401,042,909 |
| B. | Insert Length (bp) | # raw read pairs | Passed purity filter | Multiple adapters found | Incorrect Read Length | Remaining Reads |  | 101,997,106 |
| **NxTrim** | 6 kb Mate Pair | 219,631,508 | 205509152 (93.57%) | 27,784 (0.01%) | 1,487,156 (0.72%) | 203,994,212 |  |  |
|  |  |  |  |  |  |  |  |  |
|  | 8 kb Mate Pair | 273,244,682 | 245,974,730 (90.02%) | 627 (0.00%) | 35,480 (0.01%) | 245,938,623 |  |  |
|  |  |  |  |  |  |  |  |  |
|  | Insert Length (bp) | # raw read pairs | Both surviving | Forward only surviving | Reverse only surviving | Dropped | Remaining |  |
| **Trimmomatic** | 6 kb Mate Pair | 141,294,980 | 31408932 (22.23%) | 24286075 (17.19%) | 30357938 (21.49%) | 55242035 (39.10%) | 60.90% |  |
|  |  |  |  |  |  |  |  |  |
|  | 8 kb Mate Pair | 202,505,785 | 46371161 (22.90%) | 21317814 (10.53%) | 53715666 (26.53%) | 81101144 (40.05%) | 59.95% |  |
