# Supplementary material for: Genome sequence and population declines in the critically endangered greater bamboo lemur (Prolemur simus) and implications for conservation
Source: BMC Genomics. 2018 Jun 8;19:445. doi: 10.1186/s12864-018-4841-4 (PMC5994045; doi:10.1186/s12864-018-4841-4)
Supplement: Supplementary file 2 — Supplementary Materials detailing permits, genome assembly, MAKER annotation, Demographic history reconstruction- Propithecus and Microcebus, VCF Filtration. (DOCX 31 kb) [file 12864_2018_4841_MOESM2_ESM.docx]

**Supplementary Materials for Hawkins *et al.* Genome sequence and population declines in the critically endangered greater bamboo lemur (*Prolemur simus*) and implications for conservation.**

Table of Contents

[Prolemur simus permits 2](#_Toc515362853)

[Genome Assembly 2](#_Toc515362854)

[MAKER annotation 2](#_Toc515362855)

[Demographic History Reconstruction- *Propithecus* and *Microcebus* 3](#_Toc515362856)

[VCF Filtration: 4](#_Toc515362857)

[Software Development 5](#_Toc515362858)

[References 5](#_Toc515362859)

# Prolemur simus permits

USFWS and CITES permits: 601C-EA10/MG08, 08US121040/9, 601C-EA10/MG08, 08US121040/9, 01US044368/9, 01US008520/9, 370C-EA06/MG05, 05US094969/, 370C-EA06/MG05, 05US094969/9, research permits: 026/08, 227/08, 190, 181/05, 23/05, 066/10/ MEF/SG/DGF/DCB.SAP/SLRSE, 100/11/MEF/SG/DGF/ DCB.SAP/SCB, 078/12/MEF/SG/DGF/DCB.SAP/SCB, 039/13/MEF/SG/DGF/DCB.SAP/SCB

# Genome Assembly

Following testing of five different genome assemblers, we found MaSuRCA to have generated the most complete genome. This was assessed by evaluating the N50 of the contigs and scaffolds, and evaluating the recovered genes via BUSCO beta2.0. ABySS (Simpson *et al.*, 2009) recovered a total of 4,601,235 scaffolds with an N50 of 353,725 bp. ALLPATHS-LG (Gnerre *et al.*, 2010) recovered 21,558 scaffolds with an N50 of 1,779,611 bp. Platanus recovered 751,890 scaffolds with and N50 of 29,278 bp. SOAPdenovo (Luo *et al.*, 2012) recovered 246,346 scaffolds with an N50 of 331,821 bp. MaSuRCA generated 129,191 scaffolds with an N50 of 2,754,258 bp. A summary of these five assemblers with additional quality metrics are in Table 2.

# MAKER annotation

The MAKER annotation recovered: 17,548 augustus repeats; 384,277 simple repeats; 69,374 regions of low complexity; 486,142 SINE elements; 597,719 LINE elements; 2,907 unknown elements; 233,394 LTRs; 63,948 DNA/TcMar; 1,122 Helitrons; 3,365 human endogenous retroviruses; 259 HERVK elements; 565 HERVL elements; 857 satellites; 813 scRNA; 153,152 MER; 697 MULE transposons; 925 rRNAs; 111 retroposons; 33,515 Tigger transposable elements; 908 tRNAs; 1,668 snRNAs summing over two million different elements.

# Demographic History Reconstruction- *Propithecus* and *Microcebus*

Following the procedures detailed in the main text we also mapped reads from the two published reference quality lemur genomes and used those files to generate PSMC plots. The mutation rates implemented were from the mouse lemur (*Microcebus murinus*) as to our knowledge no other robust calculations are available for lemurs. Generation time was estimated at 10 years for *Propithecus* and both 3.5 and 4 years for *Microcebus* (Yoder *et al.*, 2016). The literature on age of reproduction in *Propithecus* suggests long generation time for body size, and estimates range from 6-17.5 years per generation (Richard *et al.*, 2002; Lawler, 2008; Lawler *et al.*, 2009). Less data is specifically available on wild *Microcebus* age of reproduction, but they are known to live to be 12 years old in captivity, and begin reproducing at approximately one year old. Some studies have suggested that *Microcebus* in the wild do not live more than 1-2 years, and as such we have included mutation rate estimates from published studies to avoid any biases from using a captive bred individual and inaccurate mutation rates (Yoder et al. 2016).

In the *Microcebus* reconstruction there appears to be a slow increase in the effective population size through time. In the 4.5 year generation time plot (Figure S3b) a sharp decline is shown at approximately 20,000 ybp, but as this is within the time frame where PSMC is inaccurate it is unknown if this pattern is an artifact of the analysis. The effective population size of this individual does not show a decline in either the 3 or 4.5 year generation time plots. Due to the unknown origin and captive nature of this individual we refrain from speculation. Wild-caught mouse lemurs from across Madagascar are necessary to better understand the demographic history of this genus.

The *Propithecus* PSMC plot shows a slow increase between two and one million years before present, and a slow decline beginning around one million years ago. From 50-100,000 years before present a faster decline is observed. This is also when a faster rate of decline was observed in the greater bamboo lemur. This reconstruction revealed a 53% population decline from the maximum estimate of effective population size until PSMC is inaccurate (in this case around 40,000 ybp). Additional species from within this genus will allow for through understanding of the evolution of sifakas across Madagascar.

# VCF Filtration:

VCFtools filter was used to determine the best quality variants, using a depth of greater than or equal to 10 and a map quality of greater than or equal to 40. Then once the VCF files are filtered files are reduced to those which are intersects between the five individuals and which have at least two variants. We also checked to ensure that the variants were not fixed for the alternate allele, which may be an error in the denovo assembly. Additional details can be found at: <https://gist.github.com/TheCulliganMan/e63aa129dec1f6bd6c6c9dcc0b795677> . SNVs were tested for linkage disequilibrium using vcftools (--geno-r2) on the non-thinned set of 152, 361 SNVs.

# Software Development

A variety of python scripts were written to perform operations included in this manuscript. Details of all available scripts and usage can be found at: <https://github.com/TheCulliganMan>

# References

Gnerre, S., MacCallum, I., Przybylski, D., Ribeiro, F.J., Burton, J.N., Walker, B.J., Sharpe, T., Hall, G., Shea, T.P., Sykes, S., Berlin, A.M., Aird, D., Costello, M., Daza, R., Williams, L., Nicol, R., Gnirke, A., Nusbaum, C., Lander, E.S. & Jaffe, D.B. (2010) High-quality draft assemblies of mammalian genomes from massively parallel sequence data. *Proceedings of the National Academy of Sciences*, **108**, 1513–1518.

Lawler, R.R. (2008) Testing for a historical population bottleneck in wild Verreaux’s sifaka ( *Propithecus verreauxi verreauxi* ) using microsatellite data. *American Journal of Primatology*, **70**, 990–994.

Lawler, R.R., Richard, A., Dewar, R., Schwartz, M., Ratsirarson, J. & Caswell, H. (2009) Demography of a wild lemur population in a stochastic rainfall environment. *Oecologia*, **161**, 491–504.

Luo, R., Liu, B., Xie, Y., Li, Z., Huang, W., Yuan, J., He, G., Chen, Y., Pan, Q., Liu, Y., Tang, J., Wu, G., Zhang, H., Shi, Y., Liu, Y., Yu, C., Wang, B., Lu, Y., Han, C., Cheung, D.W., Yiu, S.-M., Peng, S., Xiaoqian, Z., Liu, G., Liao, X., Li, Y., Yang, H., Wang, J., Lam, T.-W. & Wang, J. (2012) SOAPdenovo2: an empirically improved memory-efficient short-read de novo assembler. *GigaScience*, **1**, 18.

Richard, A.F., Dewar, R.E., Schwartz, M. & Ratsirarson, J. (2002) Life in the slow lane? Demography and life histories of male and female sifaka (Propithecus verreauxi verreauxi). *Journal of Zoology*, **256**, 421–436.

Simpson, J.T., Wong, K., Jackman, S.D., Schein, J.E., Jones, S.J.M. & Birol, I. (2009) ABySS: a parallel assembler for short read sequence data. *Genome research*, **19**, 1117–23.

Yoder, A.D., Campbell, C.R., Blanco, M.B., Dos Reis, M., Ganzhorn, J.U., Goodman, S.M., Hunnicutt, K.E., Larsen, P.A., Kappeler, P.M., Rasoloarison, R.M., Ralison, J.M., Swofford, D.L. & Weisrock, D.W. (2016) Geogenetic patterns in mouse lemurs (genus Microcebus) reveal the ghosts of Madagascar’s forests past. *Proceedings of the National Academy of Sciences of the United States of America*, **113**, 8049–56.
