# Supplementary figures and images for: Genome sequence and population declines in the critically endangered greater bamboo lemur (Prolemur simus) and implications for conservation
Source: BMC Genomics. 2018 Jun 8;19:445. doi: 10.1186/s12864-018-4841-4 (PMC5994045; doi:10.1186/s12864-018-4841-4)

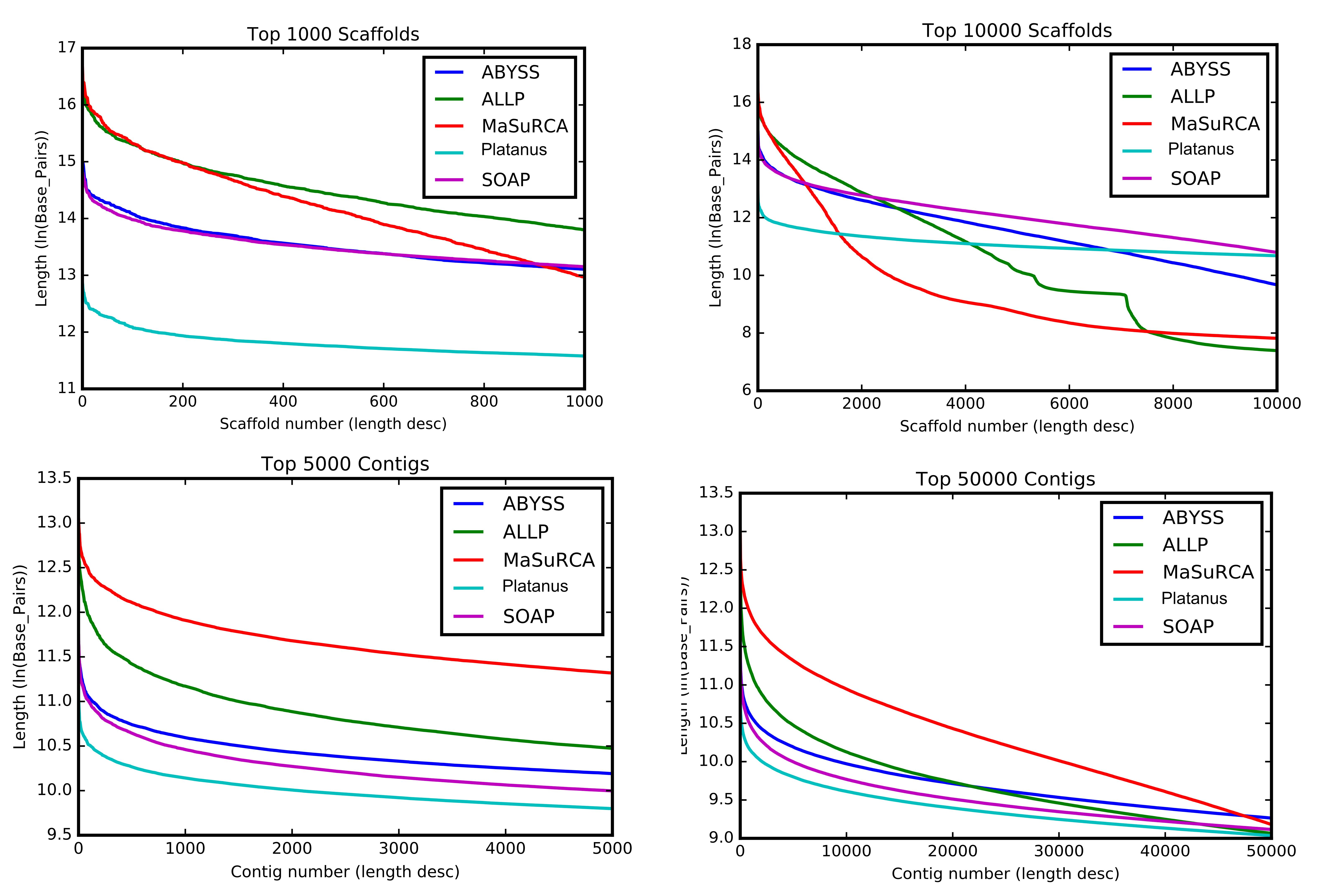

Supplement: Supplementary file 3 — Comparison of scaffolds and contigs across different assembly programs. The top row shows the largest 1000 and 10,000 scaffolds across each program (from left to right) and the bottom row displays the longest 5000 and 50,000 contigs. ALLP in the figure caption represents the ALLPATHS assembly. (JPG 3784 kb) [file 12864_2018_4841_MOESM3_ESM.jpg]

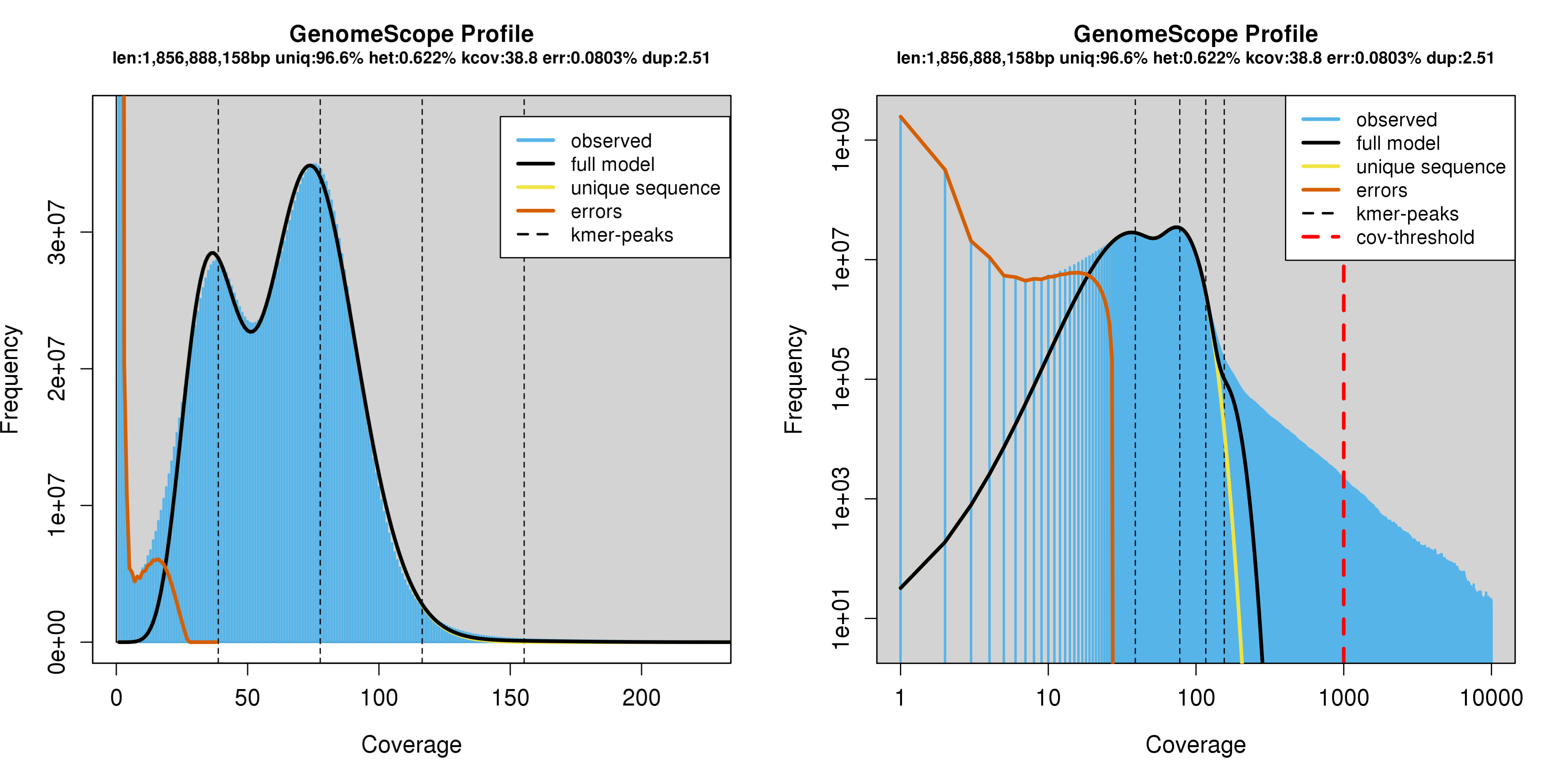

Supplement: Supplementary file 5 — GenomeScope Profile of the KIAN8.4 high coverage genome. (TIFF 2484 kb) [file 12864_2018_4841_MOESM5_ESM.tiff]

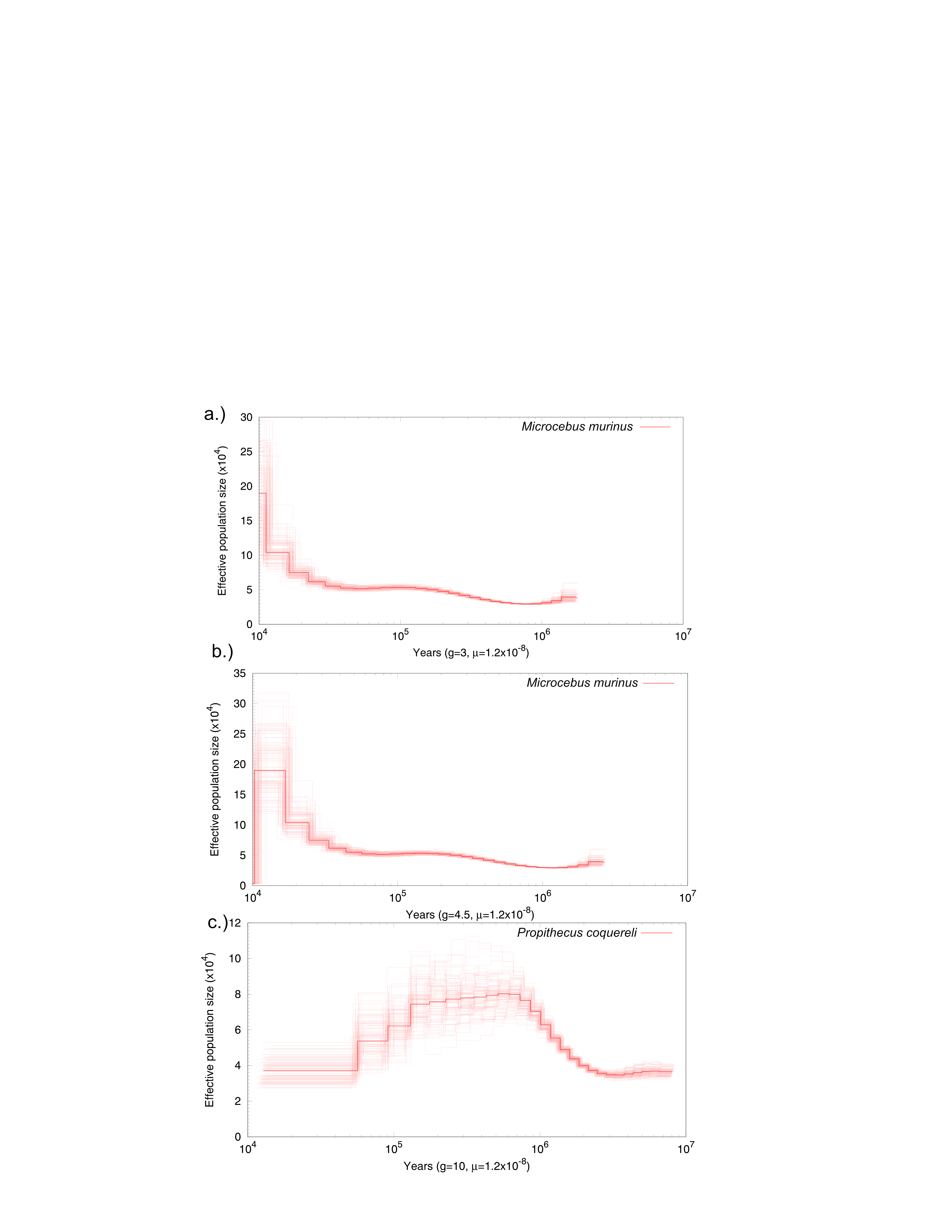

Supplement: Supplementary file 10 — PSMC plots of the two lemur reference genomes. The top two images show the Microcebus murinus genome reconstructions with 100 bootstrap replicates. Image ‘a’ applied a three-year generation time, ‘b’ incorporates a 4.5 year generation time as detailed in (Yoder et al., 2016). The third image, ‘c’, is the demographic history reconstruction of Propithecus coquereli. The population crash shown in ‘b’ occurred during the time period where PSMC reconstructions are inaccurate and as such we refrain from making inferences from that graph. (TIFF 1614 kb) [file 12864_2018_4841_MOESM10_ESM.tiff]

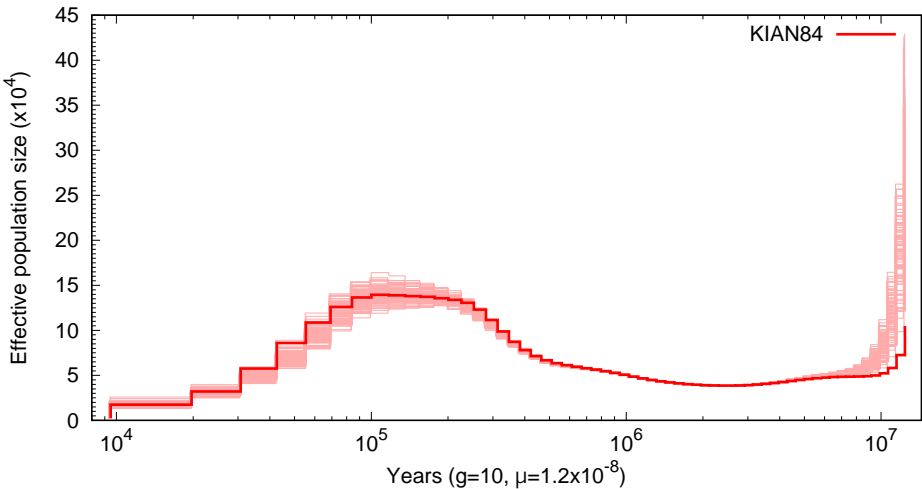

Supplement: Supplementary file 12 — PSMC plot from the 150X genome reconstruction of KIAN8.4. Note the scale of the y-axis in this graph is not the same as the PSMC plot from the main text. (PDF 174 kb) [file 12864_2018_4841_MOESM12_ESM.pdf]
