# Supplementary material for: Genome sequence and population declines in the critically endangered greater bamboo lemur (Prolemur simus) and implications for conservation
Source: BMC Genomics. 2018 Jun 8;19:445. doi: 10.1186/s12864-018-4841-4 (PMC5994045; doi:10.1186/s12864-018-4841-4)
Supplement: Supplementary file 4 — BUSCO v2.0beta assessments of genome completeness for the five different genome assemblies, using the ‘mammal’ set of 4106 genes. (DOCX 46 kb) [file 12864_2018_4841_MOESM4_ESM.docx]

Table S2. BUSCO v2.0beta assessments of genome completeness for the five different genome assemblies, using the ‘mammal’ set of 4106 genes.

| BUSCO v2.0beta | Scaffolds | |  | mammal gene set | | | | |  |
| --- | --- | --- | --- | --- | --- | --- | --- | --- | --- |
|  | Complete | Single | Multi | | Fragment | | Missing | Total recovered | % recovered |
| SOAP de novo | 3770 | 3730 | 40 | 212 | | 124 | | 3982 | 96.98% |
| ABySS | 3268 | 3251 | 17 | 503 | | 335 | | 3771 | 91.84% |
| ALLPATHS-LG | 3249 | 3220 | 29 | 435 | | 422 | | 3684 | 89.72% |
| Platanus | 2047 | 2030 | 17 | 1298 | | 761 | | 3345 | 81.47% |
| MaSuRCA | 3899 | 3868 | 31 | 113 | | 94 | | 4012 | 97.71% |
