# Supplementary material for: Genome sequence and population declines in the critically endangered greater bamboo lemur (Prolemur simus) and implications for conservation
Source: BMC Genomics. 2018 Jun 8;19:445. doi: 10.1186/s12864-018-4841-4 (PMC5994045; doi:10.1186/s12864-018-4841-4)
Supplement: Supplementary file 6 — Autosome Extraction Results from the KIAN8.4 MaSuRCA contigs. (DOCX 51 kb) [file 12864_2018_4841_MOESM6_ESM.docx]

Table S3. Autosome Extraction Results from the KIAN8.4 MaSuRCA contigs.

| Original Sequence Length | After X removal | After XY removal | After XY & WMG removal | Difference | % removed |
| --- | --- | --- | --- | --- | --- |
| 2,238,717,771 bp | 2,011,555,924 bp | 2,003,065,157 bp | 2,002,851,803 bp | 235,865,968 bp | 10.53% |
| Number of Contigs | After X removal | After XY removal | After XY & WMG removal | Difference | % removed |
| 211,576 | 202,422 | 202,121 | 202,106 | 9,470 | 1.17% |
