# Supplementary material for: Genome sequence and population declines in the critically endangered greater bamboo lemur (Prolemur simus) and implications for conservation
Source: BMC Genomics. 2018 Jun 8;19:445. doi: 10.1186/s12864-018-4841-4 (PMC5994045; doi:10.1186/s12864-018-4841-4)
Supplement: Supplementary file 7 — Quality Filtering Stats from 10X genomes using Trimmomatic v0.36. (DOCX 52 kb) [file 12864_2018_4841_MOESM7_ESM.docx]

Table S4. Quality Filtering Stats from 10X genomes using Trimmomatic v0.36.

|  | # raw read pairs | Both surviving | Forward only surviving | Reverse only surviving | Dropped | Reads retained after QC: |
| --- | --- | --- | --- | --- | --- | --- |
| KAR3 | 142,665,612 | 102026022 (71.51%) | 29878078 (20.94%) | 3099719 (2.17%) | 7,661,793 (5.37%) | 94.62% |
| KIAN8.1 | 105,812,823 | 76297730 (72.11%) | 22002226 (20.79%) | 2077916 (1.96%) | 5,434,951 (5.14%) | 94.86% |
| RANO355 | 106,950,153 | 76410319 (71.44%) | 22918501 (21.43%) | 2163774 (2.02%) | 5,457,559 (5.10%) | 94.89% |
| TORO8.24 | 106,999,021 | 80383463 (75.13%) | 19277636 (18.02%) | 2288490 (2.14%) | 5049432 (4.72%) | 95.29% |
