# Supplementary material for: Genome sequence and population declines in the critically endangered greater bamboo lemur (Prolemur simus) and implications for conservation
Source: BMC Genomics. 2018 Jun 8;19:445. doi: 10.1186/s12864-018-4841-4 (PMC5994045; doi:10.1186/s12864-018-4841-4)
Supplement: Supplementary file 8 — Statistics from mitochondrial genomes from the five Prolemur simus and one Hapalemur griseus. Coverage statistics as well as the GenBank Accession numbers are listed. (DOCX 61 kb) [file 12864_2018_4841_MOESM8_ESM.docx]

Table S5. Statistics from mitochondrial genomes from the five *Prolemur simus* and one *Hapalemur griseus*. Coverage statistics as well as the GenBank Accession numbers are listed.

|  |  | **Length** | **# Mapped Reads** | **Average Coverage** | **Min** | **Max** | **Std Dev** | **GenBank Accession #** |
| --- | --- | --- | --- | --- | --- | --- | --- | --- |
| *Prolemur simus* | KIAN8.4 | 16,853 | 136,201 | 952 | 249 | 1,238 | 141.7 | KX932098 |
| *Prolemur simus* | KIAN8.1 | 16,946 | 214,443 | 1614.4 | 356 | 2,630 | 246.8 | KX932101 |
| *Prolemur simus* | TORO8.24 | 16,949 | 229,071 | 1730.2 | 357 | 2,584 | 267.8 | KX932097 |
| *Prolemur simus* | KAR3 | 16,990 | 311,705 | 2325 | 420 | 3,558 | 410.7 | KX932099 |
| *Prolemur simus* | RANO355 | 16,965 | 177,394 | 1324.5 | 280 | 2,440 | 228.6 | KX932100 |
| *Hapalemur griseus* | DASI5.11 | 16,968 | 6,937 | 37.4 | 0 | 86 | 21.7 | KX932102 |
