# Supplementary material for: Genome sequence and population declines in the critically endangered greater bamboo lemur (Prolemur simus) and implications for conservation
Source: BMC Genomics. 2018 Jun 8;19:445. doi: 10.1186/s12864-018-4841-4 (PMC5994045; doi:10.1186/s12864-018-4841-4)
Supplement: Supplementary file 9 — Results of read mapping of published lemur reference genomes. Accession numbers, coverage, standard deviation, variants and origin of tissue are listed below. (DOCX 46 kb) [file 12864_2018_4841_MOESM9_ESM.docx]

Table S6. Results of read mapping of published lemur reference genomes. Accession numbers, coverage, standard deviation, variants and origin of tissue are listed below.

| **Reference Genome** | **GenBank Accession #** | **Average Coverage** | **Standard Deviation** | **Raw Data Used:** | **# variants** | **Tissue source** |
| --- | --- | --- | --- | --- | --- | --- |
| *Propithecus coquereli* | SRX763490 | 8.83 | 26.57 | SRR1657028 | 6,614,286 | Kidney |
| *Microcebus murinus* | SRX767314 | 12.44 | 31.07 | SRR1662129 | 6,808,109 | Kidney |
