# Supplementary material for: Genome sequence and population declines in the critically endangered greater bamboo lemur (Prolemur simus) and implications for conservation
Source: BMC Genomics. 2018 Jun 8;19:445. doi: 10.1186/s12864-018-4841-4 (PMC5994045; doi:10.1186/s12864-018-4841-4)
Supplement: Supplementary file 11 — PSMC estimates of effective population size following Pleistocene bottleneck. (DOCX 35 kb) [file 12864_2018_4841_MOESM11_ESM.docx]

Table S7. PSMC estimates of effective population size following Pleistocene bottleneck.

| **ID** | | **Estimated Population** |
| --- | --- | --- |
| KIAN8.4 | 126,212 | |
| KIAN8.1 | 95,663 | |
| TORO 8.24 | 169,227 | |
| RANO355 | 61,897 | |
| KAR3 | 92,446 | |
| *Average:* | *109,099* | |
