# Supplementary material for: Genome sequence and population declines in the critically endangered greater bamboo lemur (Prolemur simus) and implications for conservation
Source: BMC Genomics. 2018 Jun 8;19:445. doi: 10.1186/s12864-018-4841-4 (PMC5994045; doi:10.1186/s12864-018-4841-4)
Supplement: Supplementary file 13 — A comparison of the effect of buffered edges around the 1950’s forest cover. The minimum convex polygon is shown in pale yellow, and the three different buffered distances are shown in light blue, peach and olive respectively. The degree of deforestation would affect which buffered distance is most likely for this species to have historically occupied. (PDF 5673 kb) [file 12864_2018_4841_MOESM13_ESM.pdf]

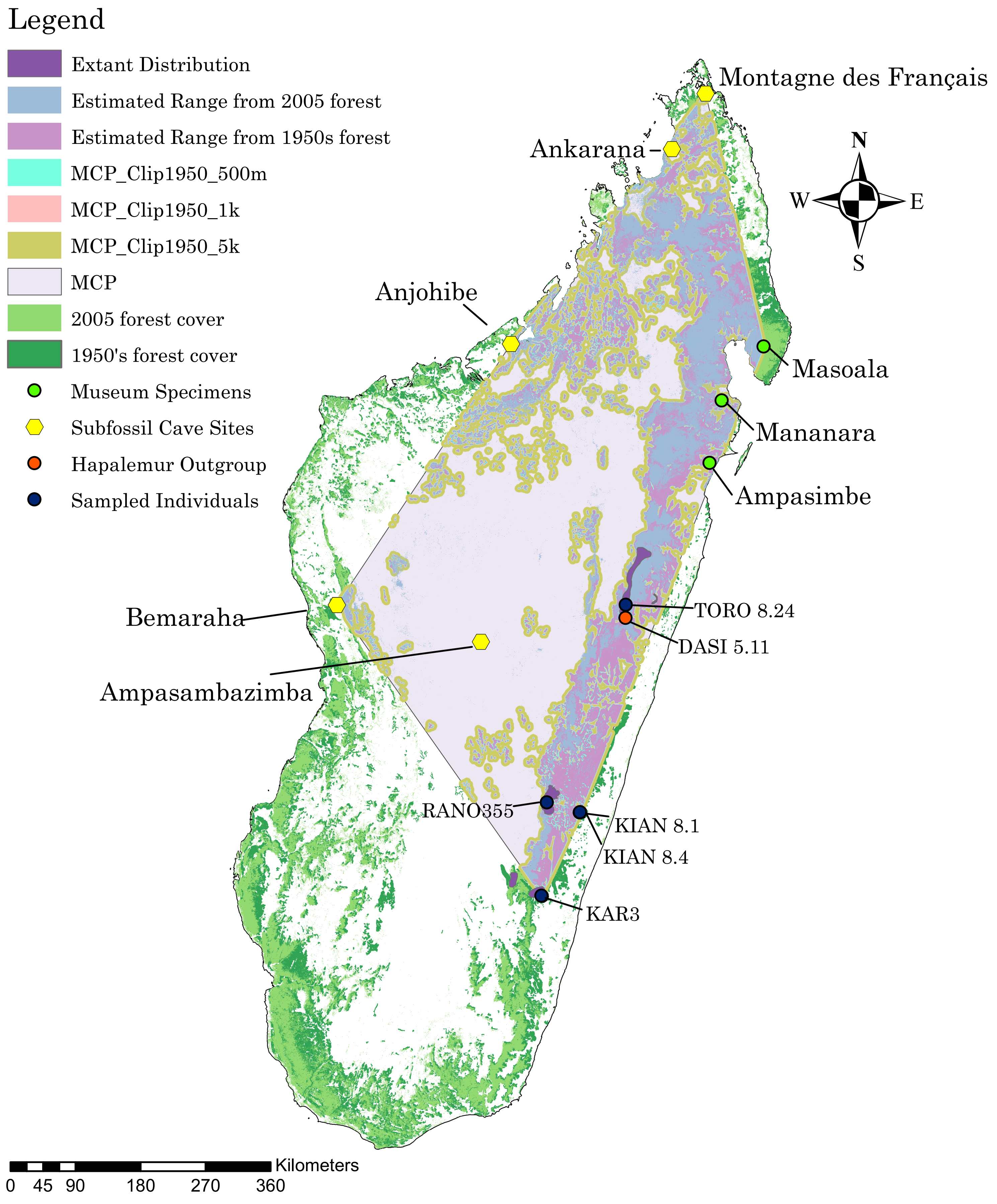

Legend
Extant Distribution
Estimated Range from 2005 forest
Estimated Range from 1950s forest
MCP_Clip1950_500m
MCP_Clip1950_1k
MCP_Clip1950_5k
MCP
2005 forest cover
1950's forest cover
Museum Specimens
Subfossil Cave Sites
Hapalemur Outgroup
Sampled Individuals
Montagne des Français
Ankarana
N
W
E
S
Anjohibe
Masoala
Mananara
Ampasimbe
TORO 8.24
DASI 5.11
Bemaraha
Ampasambazimba
RANO355
KIAN 8.1
KIAN 8.4
KAR3
Kilometers
0
45
90
180
270
360
