# Supplementary material for: Genome sequence and population declines in the critically endangered greater bamboo lemur (Prolemur simus) and implications for conservation
Source: BMC Genomics. 2018 Jun 8;19:445. doi: 10.1186/s12864-018-4841-4 (PMC5994045; doi:10.1186/s12864-018-4841-4)
Supplement: Supplementary file 14 — Historical range calculations based on varying levels of forest buffer. Forest area is given in square kilometers. (DOCX 39 kb) [file 12864_2018_4841_MOESM14_ESM.docx]

Table S8. Historical range calculations based on varying levels of forest buffer. Forest area is given in square kilometers.

| **Occupied forest estimates:** |  |  |
| --- | --- | --- |
| 1711.74 | current distribution | % remaining |
| 44259.65 | 2005 | 3.87% |
| 80375.37 | 1950s | 2.13% |
| 96,144.10 | 500 m buffer added | 1.78% |
| 108,003.45 | 1 km buffer added | 1.58% |
| 163,223.84 | 5 km buffer added | 1.05% |
